# A pivotal role of the vascular endothelial growth factor signaling pathway in the formation of venous hypertension-induced dural arteriovenous fistulas

**DOI:** 10.3892/mmr.2014.2037

**Published:** 2014-03-11

**Authors:** QIANG LI, QI ZHANG, QING-HAI HUANG, YI-BIN FANG, ZHAO-LONG ZHANG, YI XU, JIAN-MIN LIU

**Affiliations:** Department of Neurosurgery, Changhai Hospital, Second Military Medical University, Shanghai 200433, P.R. China

**Keywords:** dural arteriovenous fistula, vascular endothelial growth factor, angiogenesis, venous hypertension, local hypoferfusion

## Abstract

Dural arteriovenous fistulas (DAVFs) are associated with venous hypertension. Numerous studies have revealed high expression levels of vascular endothelial growth factor (VEGF) in human DAVF specimens, as well as in animal models of experimental venous hypertension. The objective of the present study was to clarify whether the VEGF signaling pathway is important in the development of DAVFs. Rats (n=216) were randomly divided into six groups. In the rats from five groups (groups A and C-E, n=45 in each group; group B, n=12), experimental venous hypertension was induced by right common carotid artery (CCA)-external jugular vein (EJV) anastomosis, superior sinus occlusion and left transver sinus occlusion, while the remaining group (group F, n=24) underwent sham surgery. The rats in group A received a VEGF recombinant adenovirus injection into the distal section of the right EJV 30 min prior to anastomosis of the CCA and EJV. An equivalent control adenovirus was injected into the right EJV of group B rats prior to anastomosis. The rats in group C received no virus prior to anastomosis and no medicine subsequent to surgery. The group D rats were lavaged with Vatalanib, a VEGF receptor (VEGFR) inhibitor, and the group E rats were lavaged with an equal quantity of saline weekly following surgery. Six rats from groups A-E and one rat from group F were sacrificed in the first, second, fourth and twelfth weeks after surgery for immunohistochemical analysis of VEGF expression and analysis of microvessel density. Cerebral angiography was performed on the remaining rats in each group on the twelfth week after surgery. The results revealed that following transfection with VEGF recombinant adenovirus, angiogenesis in the dura mater of venous hypertensive rats was increased subsequent to the increase in the VEGF expression levels of the brain and dura mater. The rate of DAVF induction by venous hypertension was significantly reduced by the VEGFR antagonist due to reduced angiogenesis in the dura mater. In conclusion, VEGF and its receptor may be important in the formation of venous hypertension-induced DAVFs.

## Introduction

Dural arteriovenous fistulas (DAVFs) are pathological arteriovenous shunts that involve any section of the dura mater and its adjacent structures, and account for 10–15% of all intracranial arteriovenous malformations (1). Although the etiopathogenesis remains unclear, DAVFs are considered to be acquired lesions, in contrast to arteriovenous malformations (1,2).

Venous sinus hypertension, which may have multiple etiologies, is hypothesized to predispose patients to DAVFs (3). DAVFs can be developed in rats by surgically inducing venous hypertension via common carotid artery (CCA)-external jugular vein (EJV) anastomosis, followed by draining venous or dural sinus occlusion (4). Lawton *et al* (5) observed angiogenesis of the sclera and corneal limbus following implantation of dura mater tissue (obtained from rats with chronic venous hypertension at different time periods) into rabbit corneas. In addition, high expression of vascular endothelial growth factor (VEGF) and other angiogenic growth factors has been detected in clinical DAVF samples (6,7). These studies suggest that a high expression of angiogenic factors, including VEGF, may be associated with the formation of DAVFs. However, it remains unknown whether the high expression of VEGF is pivotal in the formation of DAVFs or is only a secondary effect. The purpose of the present study was to observe angiogenesis in the dura mater and cerebral cortex, and the formation of DAVFs in venous hypertension models when VEGF/VEGF receptor (VEGFR) signaling pathways are modified. Activation and inhibition of the VEGFR pathways was achieved using VEGF recombinant adenovirus and a VEGFR inhibitor, respectively, in order to clarify the role of the VEGF/VEGFR signaling pathways in the formation of venous hypertension-induced DAVFs.

## Materials and methods

### Animal studies

All experiments involving animals were approved by the Institutional Animal Care and Use Committee of Changhai Hospital (Shanghai, China). To exclude any potential hormonal effects on DAVF formation, only male Sprague-Dawley rats, weighing 350–400 g, were used. All rats were raised and maintained under standard laboratory conditions.

### Grouping and operation

All rats underwent surgical procedures following intraperitoneal injection of 10% chloral hydrate at a dose of 0.5 ml/100 g body weight. The injection was supplemented as required during the procedure. All surgical procedures were performed using standard sterile techniques. A total of 192 rats underwent right CCA-EJV anastomosis, followed by superior sagittal sinus ligation and left transverse sinus (facial vein) occlusion. The other 24 rats received sham surgery.

A modification of the model described by Chen *et al* (8) was used. A frontal median incision was made and the skull was drilled through to expose the superior sagittal sinus and this was ligated with the use of a no. 10-0 polypropylene suture (Ethicon, Cincinnati, OH, USA). Through a second incision below the left ear, the left terminal end of the transverse sinus was exposed and destroyed using bipolar electrocoagulation. Through a cervical median incision, the right anterior facial vein was ligated with a no. 10-0 polypropylene suture and the proximal CCA was anastomosed to the distal EJV in an end-to-end manner using no. 10-0 polypropylene interrupted sutures. This resulted in retrograde flow through the transverse sinus. The proximal segment of the EJV and the initial portions of the right external and internal carotid arteries were separately destroyed. Subsequent to surgery, the incision was ligated with a no. 3-0 polyglactin suture (Ethicon). The rats that received sham surgery only underwent frontal, post-aurem, and cervical medial incision, and suture.

All rats were randomly divided into six groups immediately prior to the procedures. Group A included 45 rats that underwent superior sagittal sinus and left transverse sinus occlusion, isolation and dissection of the right EJV. They were then injected with VEGF recombinant adenovirus (3×10^9^/plaque-forming unit, diluted to 0.3 ml) in the distal right EJV. The EJV was then anastomosed to the CCA in an end-to-end manner following occlusion for 30 min. Group B comprised 12 rats that underwent the same surgical procedure as in group A, but were injected with an equivalent quantity of control adenovirus instead of VEGF recombinant adenovirus. Groups C, D and E (n=45 rats per group) underwent the same surgical procedure as group A, but without injection of any adenovirus. Following surgery, the rats in groups A, B and C were fed according to routine methods. The group D rats were lavaged with the VEGFR inhibitor, vatalanib (PTK787/ZK222584, 5 mg/100 g of body weight; LC Labs, Woburn, MA, USA) and the group E rats were lavaged with an equal quantity of saline weekly, prior to and subsequent to surgery. The 24 rats in group F that received sham surgery served as a control group.

### Construction of VEGF recombinant adenoviral vectors

A VEGF165 gene fragment was amplified using polymerase chain reaction (PCR), digested with *Eco*RI and *Sal*I (Invitrogen, Carlsbad, CA, USA), then connected onto the plasmid carrier (pDC316-mCMV; Minghong Biological Engineering Co., Ltd., Shanghai, China) for construction of a shuttle plasmid (pDC316-VEGF165). A total of 293 cells were transfected with the shuttle plasmid, skeleton plasmid (pBHGlox_E1,3Cre) and an adenovirus packaging system (AdMax^™^, Microbix Biosystems Inc., Mississauga, ON, Canada). The cells were transfected with the first generation of virus (amplified by cell culture) to obtain the second generation of the virus, which was identified using PCR. Another 293 cells were transfected with the second generation of the virus in order to gain a large number of virus particles from multiple cultures. SOURSE 15Q ion-exchange purification (GE Healthcare Life Sciences, Piscataway, NJ, USA), molecular sieve purification and desalination were performed. The purified virus was filtered to remove bacteria.

### Histological and microvascular density (MVD) examination

At 1, 2, 4 and 12 weeks after surgery (only at 1 and 2 weeks in group B), six rats from groups A-E and one rate from group F were anesthetized with an intraperitoneal injection of 7% chloraldurate (0.5 ml/100 g body weight) and perfused intracardially with 0.9% saline followed by fresh 4% paraformaldehyde in 0.1 mol/l phosphate buffer (pH 7.2). The brains of the rats, along with the dura mater, including the dural sinuses, were carefully removed and postfixed for 48 h in 4% paraformaldehyde. Sections of dura mater and brain tissue (1×1×0.5 cm) around the torcular herophili area were harvested. The dura mater on the surface of the brains were fully unfolded and packed with gauze, rinsed under running water, dehydrated with a set of varying alcohol concentrations, immersed in a mixture of equal quantities of xylene and dehydrated ethanol for 1 h, and hyalinized in pure xylene for 20 min. The masses were embedded with paraffin (melting point, 45–50°C) for 30 min. The paraffin blocks were transected perpendicular to the superior sagittal sinus at a thickness of 8 μm.

The tissue biopsy slides were immersed in dimethylbenzene for dewaxing (subsequent to baking in a drying oven at 56°C for 1.5 h) and then immersed in 100, 90 and 70% ethanol in turn for 3 min each. The slides were washed in phosphate-buffered saline (PBS; 0.01 mol/l, pH 7.2) for antigen retrieval, immersed in boiling citric acid buffer (0.01 mol/l, pH 6.0) for 15 min, cooled at room temperature following heat preservation, incubated in 0.3% H_2_O_2_ for 20 min at room temperature and then incubated in 10% normal fetal bovine serum for 30 min at room temperature. The sections were rinsed with PBS and then incubated overnight with either rabbit monoclonal primary antibody against VEGF (Abcam, Cambridge, MA, USA, 1:200) or rabbit monoclonal primary antibody against CD31 (Abcam, 1:150) in PBS containing 1% normal goat serum and 0.3% Triton X-100 at 4°C. The sections were washed three times in PBS for 3 min each and then incubated for 1.5 h at 37°C with horseradish peroxidase-conjugated goat anti-rabbit antibody (Jackson ImmunoResearch Laboratories, Inc., Westgrove, MA, USA), diluted to 1:200 in PBS containing 1% normal goat serum and 0.3% Triton X-100. The slides were rinsed with PBS three times for 3 min, stained with 3′3′-Diaminobenzidine for 3–5 min, restained with hematoxylin for 30 sec and differentiated with alcohol containing 1% hydrochloric acid for 1 sec. For dehydration, the slides were immersed in 70, 90 and 100% ethanol in turn for 3 min each. The slides were then dried, wax-sealed and mounted.

The immunohistochemical results were analyzed using Image-Pro Plus 6.0 (Media Cybernetics, Bethesda, MD, USA). Yellow or brown granules were regarded as positive signals. The expression level of VEGF was presented as the positive area ratio. The high expression areas in the occipital cortex and dura mater were identified in the VEGF staining slices using adjacent sections of the tissues from each time point at a low power (magnification, ×40) and then high power (magnification, ×200). Three high expression areas in the occipital cortex and dura mater were randomly selected for calculation of the VEGF expression positive area ratio. The MVD was defined as the CD31-positive microvascular number per unit area on the slice. Adjacent sections of the slices with CD31 staining from groups A, C, D, E and F were observed at 4 and 12 weeks at high power (magnification, ×400), and the microvascular number was recorded in three high-power fields in the dura. The dural MVD was shown as the microvascular number per square millimeter, which was converted from the mean of three microvascular numbers.

### Angiographic experiments

All angiography was conducted using a Innova 2100-IQ (GE Healthcare, New York, NY, USA). Cerebral angiography was performed 12 weeks after initial surgery on the remaining rats in groups A, B, C, D, E and F. All surgical procedures were performed using standard sterile techniques. Appropriate anesthesia was induced in each animal via intraperitoneal injection of 7% chloraldurate (0.5 ml/100 g body weight). The previous cervical median incision was reopened and the right CCA and EJV were exposed to confirm the patency of the right CCA-EJV anastomosis. The rats with occluded anastomosis were excluded from angiographic analysis. Following the ligation of the CCA-EJV bypasses with no. 3-0 polyglactin sutures, the abdominal center was opened, the abdominal aorta was dissociated, the distal section was ligated and a 4 French arterial sheath (Terumo Corporation, Tokyo, Japan) was placed into the proximal section. The left CCA was selected with a 3 French microcatheter guided by a microguide wire and 2 ml iopromide (diluted at 1:1 with normal saline) was injected using a high-pressure syringe at 0.6 ml/s. Anteroposterior and lateral angiography was continuously performed at 6 frames/sec until the venous sinus period was reached. Dynamic images were analyzed by three neurointerventional physicians; DAVF formation was confirmed when either the intracranial cortical vein or the venous sinus was enhanced in the arterial phase.

### Statistical analysis

All values in the text, tables and figures are presented as the mean ± standard deviation. Analysis of variance was used to examine the differences among groups. When the difference was significant, the least significant difference and Student-Newman-Keuls post hoc tests were used for multiple comparisons to identify which group differences had significant P-values. For cerebral angiography, the differences among groups were analyzed by the Pearson’s χ^2^ test or Fisher’s exact test, as appropriate. The statistical analyses were conducted using SPSS 18.0 software (SPSS, Inc., Chicago, IL, USA) and P<0.05 was considered to indicate a statistically significant difference.

## Results

### VEGF expression

**Figure 1:**
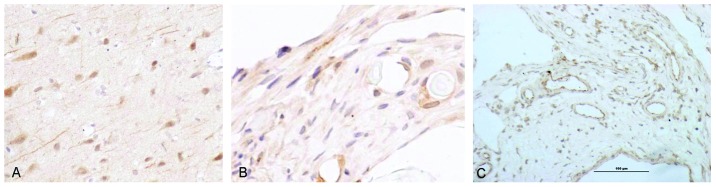
(A) Immunohistochemical staining of vascular endothelial growth factor (VEGF) in the brain tissue 2 weeks after surgery in group C (magnification, ×200). VEGF is mainly expressed in small blood vessel walls, the vascular matrix, neurons and glial cells. (B) Immunohistochemical staining of VEGF in the dura mater 2 weeks after surgery in group C (magnification, ×400). VEGF is mainly expressed in small blood vessels, endothelial cells and the vascular matrix. (C) CD31 staining at 4 weeks after surgery in group A rats (magnification, ×200) reveals significant microvascular hyperplasia in the dura mater. Staining, immunohistochemical techniques and diaminobenzidine (DAB) staining. Yellow or brown granules on the sections were recognized as positive.

VEGF was identified in all groups to be predominantly expressed in the vascular matrix of small blood vessels, endothelial cells, the vascular matrix of the dura mater, and the neurons and glial cells of the occipital lobe cortex (Fig. 1).

**Figure 2:**
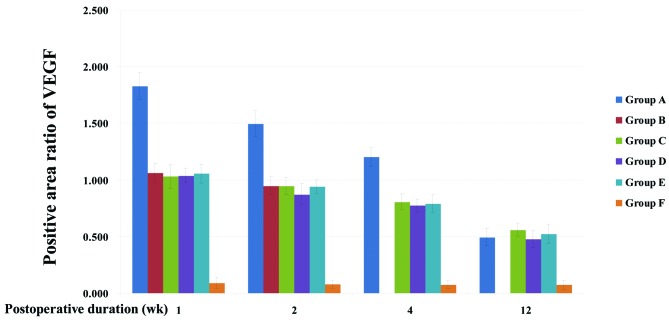
Positive area ratio of vascular endothelial growth factor expression in the cortex.

**Figure 3:**
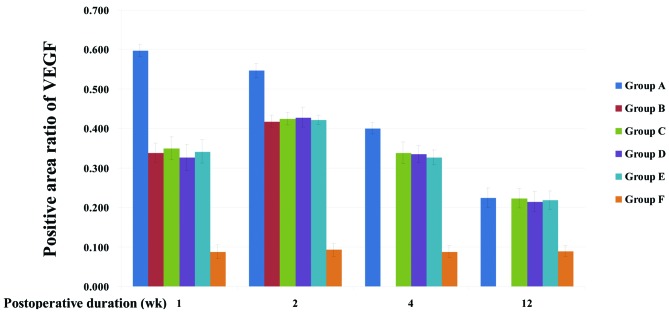
Positive area ratio of vascular endothelial growth factor expression in the dura mater.

Group A, C, D and E rats exhibited continued high expression of VEGF in the occipital cortex and dura mater from the first week after surgery to the end of the study period. This was significantly higher than the expression of VEGF in group F (P<0.001), which was low. The expression levels of VEGF at 1, 2 and 4 weeks after surgery in group A rats were significantly higher than those of groups B, C, D and E (P<0.001). In group A rats, VEGF was most highly expressed in the occipital cortex and dura mater the first week after surgery. This expression declined gradually, and no significant difference compared with the expression levels in groups C, D and E was identified 12 weeks after surgery (P>0.05). In the dura mater and cerebral cortex, no significant difference in VEGF expression levels was identified among groups B, C, D and E at any time point. In group B, C, D and E rats, peak expression of VEGF in the occipital cortex was observed in the first week after surgery (P<0.001), while in the dura mater, peak expression was observed 2 weeks after surgery (P<0.001). The expression level decreased 4–12 weeks after surgery (Tables I and II; Figs. 2 and 3).

### MVD assay

Dural capillary hyperplasia was observed 4 weeks after surgery in groups A, C, D and E (Fig. 1). The MVD of rats in groups A, C, D and E was significantly higher than that of rats in group F (P<0.01). The MVD in the dura mater was 349±24/mm^2^ in group A 4 weeks after surgery, significantly higher than that of group C (274±15/mm^2^, P<0.001), and the MVD in the dura mater was 242±13/mm^2^ in group D 4 weeks after surgery, significantly lower than the MVDs of group C (P<0.001) and group E (264±20/mm^2^, P<0.05).

**Figure 4:**
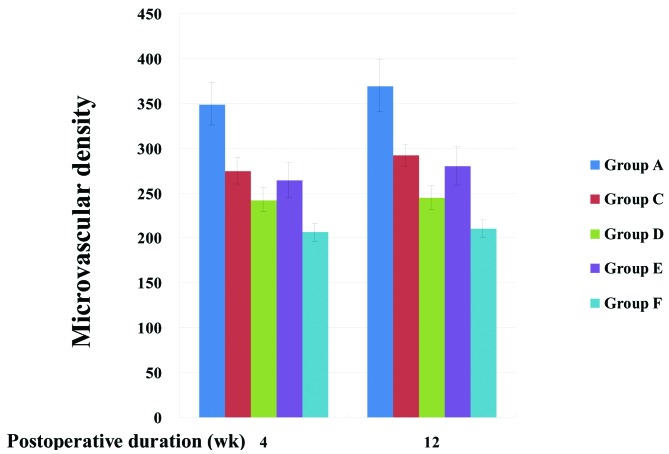
Microvascular density in the dura mater at 4 and 12 weeks.

The MVDs in the dura mater 12 weeks after surgery were 369±29/mm^2^, 291±13/mm^2^ and 280±22/mm^2^ in groups A, C and E, respectively, indicating an increase when compared with those at 4 weeks. However, there were no significant differences identified between the MVDs at 4 weeks and 12 weeks (independent samples t-test, P>0.05). The MVD in the dura mater at 12 weeks was 245±13/mm^2^ in group D, similar to that at 4 weeks. The MVD of group A at 12 weeks was significantly higher than that of group C (P<0.001), a similar result to that at 4 weeks, while the MVD of group D at 12 weeks was significantly lower than those of group C (P<0.001) and group E (P<0.01, Fig. 4).

### DAVF formation

Twelve weeks after surgery, the cervical incisions of all groups were opened prior to radiography and CCA-EJV anastomosis occlusion was identified in one rat in group A and one in group E. Angiography was successfully performed on all the remaining 102 rats and the images were clear enough for identification.

**Figure 5:**
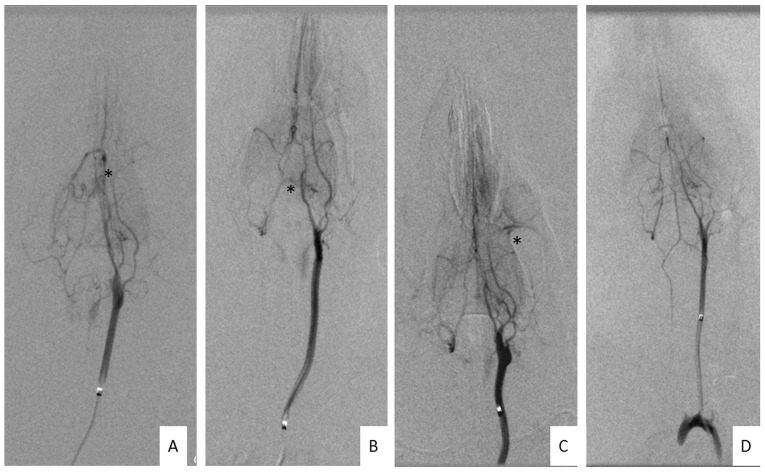
Angiography of the left common carotid artery. Asterisks show the locations of dural arteriovenous fistulas (DAVFs). (A) DAVF in the sagittal sinus. (B) DAVF in the transverse sinus. (C) DAVF in the basis cranii. (D) Angiography of normal rats.

DAVF formation occurred in 13 of the 20 rats in group A, 9 of the 21 rats in group C, 2 of the 21 rats in group D, 10 of the 20 rats in group E and none of the 20 rats in group F. Of the 34 DAVFs, 9 were located in the superior sagittal sinus area, 15 in the transverse-sigmoid sinus area and 10 in the basis cranii (Fig. 5).

The DAVF induction rate in group A (65.0%) was not identified to be significantly different from that of group C (42.9%, P=0.155). However, the DAVF induction rate of group D (9.5%) was significantly lower than those of groups C (P=0.035) and E (50.0%, P=0.012), with no significant differences identified compared with that of group F (P=0.488). According to these results, venous hypertensive rats with VEGF recombinant adenovirus transfection exhibited higher levels of VEGF expression and MVD. However, the DAVF induction rates were not significantly higher than the rates of venous hypertensive rats without adenovirus transfection. The DAVF formation rates decreased significantly in rats treated with VEGFR antagonists.

## Discussion

Clinical studies suggest that DAVFs result from intracranial venous sinus thrombosis and stenosis (9,10). Certain studies have reported that 39–80% of DAVF patients had intracranial venous sinus thrombosis and some other DAVF patients had venous sinus dysplasia, stenosis or separation, instead of thrombosis. (9,10). However, numerous cases of DAVFs without venous sinus abnormalities have also been reported.

DAVFs have been established in a venous hypertensive model, which has confirmed that venous sinus hypertension is one of the main causes of DAVFs. In 1994, Terada *et al* (4) focused on DAVF etiology in the chronic sinus hypertensive rat model. Anastomosis was performed between the right CCA and the EJV, and successfully induced DAVFs. One year later, Herman *et al* (11) improved this method through embolization of the lateral transverse sinus and superior sagittal sinus, which further increased venous hypertension, and the DAVF induction rate reached 40%.

In the present study, all the extracranial lateral branches, including the anterior facial veins in rats of groups A, B, C, D and E, were ligated prior to suturing the right EJV with the CCA, which reduced venous collateral circulation and further increased postoperative venous hypertension. According to Chen *et al* (8), the postoperative venous pressure can reach >20 mmHg in >80% of rats. Although venous collateral circulation can be established with time and can return to baseline levels within 2–4 weeks, the cerebral angiogram in groups C and E at 12 weeks revealed that the DAVF induction rate was as high as 50%.

The mechanism underlying how venous hypertension induces DAVFs remains unknown. In 1997, Lawton *et al* (5) first hypothesized that angiogenesis was involved. The dura mater of venous hypertensive rats was implanted into rabbit corneas, which exhibit extremely active angiogenesis, and DAVF formation was found to positively correlate with sinus hypertension and rabbit corneal angiogenic activity. Based on these results, it was hypothesized that venous hypertension decreases cerebral perfusion and produces brain ischemia. Tissue hypoxia subsequently stimulated angiogenesis in order to reverse the ischemia, while the increased angiogenesis of the dura mater led to the formation of arteriovenous shunts and the formation of DAVFs.

VEGF is one of the most important angiogenic factors, which can promote angiogenesis and proliferation of vascular endothelial and vascular smooth muscle. The overexpression of VEGF and other factors associated with angiogenesis has been detected in clinical DAVF specimens by Tirakotai *et al* (6) and Uranishi *et al* (7). Similar results were also observed in venous hypertensive rat models (8), suggesting that VEGF may be important in the process of venous hypertension-induced DAVF formation.

Chen *et al* (8) investigated the cerebral blood flow in venous hypertensive rats using Doppler ultrasound and found that chronic hypoperfusion around the right occipital lobe occurred in rats that had undergone right CCA-EJV anastomosis and occlusion of the contralateral transverse sinus and the superior sagittal sinus. The cerebral blood flow in the right occipital lobe immediately declined following surgery; although it continuously improved during the postoperative period, it remained 11.44% lower than preoperative levels at 12 weeks, suggesting that venous hypertension can lead to chronic, stable, local hypoperfusion (8).

Hypoxia inducible factor (HIF) responds rapidly to the venous sinus hypertension-induced ischemic state. Following induction of venous hypertension, endothelial cells in the venules beside the sagittal sinus were identified to immediately exhibit high expression of HIF-1, up to 5 times greater than the control group, with peak levels one day after surgery (12,13).

Hypoxia is one of the most important factors promoting VEGF expression. High VEGF expression occurs in intracranial tissues with cerebral hypoperfusion or hypoxia. Shin *et al* (14) observed high VEGF expression in vascular endothelial cells and connective tissues in one-third of rats 1 week after venous hypertension establishment. Zhu *et al* (12) also found that VEGF expression peaked in the basal ganglia region and the glial cells of the cerebral cortex 1 week after the venous hypertensive model was established, similar to the results of Chen *et al* (8). Chen *et al* (8) also observed that following establishment of venous hypertension, VEGF was highly expressed in the cytoplasm and vascular matrix of epidural vascular endothelial cells, peaking in the second week, and remaining strongly positive at 12 weeks. In accordance with the results of these previous studies, the present study observed that rats with venous hypertension (group C) exhibited high expression of VEGF in the occipital cortex 1 week after model induction. The VEGF levels gradually declined, but remained significantly higher than those of the control group at 12 weeks after surgery. VEGF expression in the dura mater near the sinus reached its peak at 2 weeks after surgery.

It remains unclear whether the high expression of VEGF around the DAVF lesions is key to DAVF formation or whether it is only an accompanying phenomenon. A study revealed that physiological arteriovenous shunts are in conformity with respect to the location and structure of the fistula formation (15). These results may suggest that DAVFs are induced by the opening of physiological arteriovenous shunts, but not by angiogenesis.

The aim of the present study was to clarify the role of VEGF/VEGFR. Analyses were conducted to identify whether increased local levels of VEGF improved the induction of DAVFs. Studies on ischemic cerebrovascular diseases revealed that the expression of VEGF in a target region can be effectively increased by VEGF165 adenovirus and plasmid transfection; the expression peaks ~1 week after transfection, similar to that of endogenous VEGF expression in venous hypertensive rats (16). In the current study, VEGF was overexpressed in venous hypertensive rats using venous sinus perfusion of VEGF recombinant adenovirus. VEGF was highly expressed in the brain cortex and dura mater of rats in group A, a similar location to the expression in groups B and C. Moreover, the VEGF expression levels at 1 and 2 weeks after surgery in group A rats were significantly higher than those of rats in groups B and C. These levels gradually declined 4 weeks later, and eventually declined to a level similar to that observed in venous hypertensive rats without VEGF recombinant adenovirus transfection (group C) at 12 weeks. These results suggest that VEGF can be successfully increased in the highly expressed VEGF regions of venous hypertensive rats by recombinant adenovirus, resulting in a short-term increase in VEGF levels.

At 4 and 12 weeks after surgery, active vascular proliferation in the dura was observed in the rats of the venous hypertension group (group C) and the MVD levels were significantly higher than those of the control group (group F). Markedly greater active angiogenesis was identified in the group A rats compared with those in group C, which demonstrated that VEGF levels further increase with recombinant adenovirus transfection. Although angiography suggested that the rate of DAVF induction in venous hypertensive rats with adenovirus transfection was higher than that of the simple venous hypertension group (groups C and E), no significant difference was identified, which may be due to the relatively small sample size. Conversely, this also suggests that the angiogenesis mediated by VEGF and its receptor is not the only factor that affects DAVF formation. Certain new blood vessels retrograde following formation, while others mature since they arise from the vascular basement membrane, a process termed vessel remodeling (17). In the process of DAVF formation, whether abnormal blood vessel hyperplasia is complicated by vascular remodeling requires further validation. In addition, other factors associated with angiogenesis, such as Ephrin (18) and metal matrix proteases (8), are also important in the process of angiogenesis and vascular remodeling. As a result, improving VEGF expression levels alone may not be able to significantly increase the rate of DAVF induction.

However, the current study has identified that vascular hyperplasia of the dura and brain cortex in venous hypertensive rats markedly declined using the VEGF receptor antagonist Vatalanib, a type of tyrosine kinase inhibitor of VEGFR that inhibits VEGF/VEGFR signaling pathways by competitively interfering with VEGF binding sites on VEGFR and subsequently affecting angiogenesis (19–20). The DAVF induction rates were also significantly decreased following vatalanib administration, suggesting that the effect of venous hypertension on DAVF induction can be inhibited by VEGFR antagonism. Therefore, the results of the current study illustrate that VEGF/VEGFR is key in the process of venous hypertension-induced DAVF formation. However, DAVFs were still observed in certain rats. Further studies are required to determine whether this result was observed due to Vatalanib drug concentrations or as a result of other factors in venous hypertension-induced DAVF formation, including the opening of physiological arteriovenous shunts and the series of changes in the biological characteristics of the vascular endothelial cells in cerebral veins and venous sinuses due to venous hypertension (21).

The sample size of the current study was limited due to the difficulty in establishing a venous hypertensive rat model; therefore, this study was preliminary research. The adenovirus-transfected region may have been more clearly identified if the adenovirus injected in the control group (group B) had been replaced with adenovirus with green fluorescence labeling. In addition, all the VEGF expression data were obtained by semi-quantitative immunohistochemical analysis, but western blotting may have been more accurate for the determination of cytokines. However, in order to obtain brain tissues with dura, the tissues were fixed with paraformaldehyde prior to euthenizing the rats, rendering the samples unsuitable for western blotting. Finally, due to the small sample size, the optimum dosage of vatalanib was not investigated. Therefore, the efficiency of vatalanib-induced inhibition of the VEGF/VEGFR pathway activated by venous hypertension may not have reached its maximum.

In conclusion, angiogenesis in the dura mater of venous hypertensive rats was increased by transfection with VEGF recombinant adenovirus, subsequent to increases in the VEGF expression levels in the brain and dura mater. However, the induction rate of DAVF induced by venous hypertension was significantly reduced using a VEGFR antagonist due to reduced angiogenesis in the dura mater. These results suggest that activation of the VEGF/VEGFR signaling pathway is important in the formation of venous hypertension-induced DAVFs.

## Figures and Tables

**Table I:** Positive area ratio of vascular endothelial growth factor expression in the cortex.

Group	1 week	2 weeks	4 weeks	12 weeks
A	1.828±0.119^a^	1.498±0.112^a^	1.203±0.084^a^	0.495±0.078
B	1.062±0.082	0.950±0.078		
C	1.032±0.108	0.947±0.077	0.808±0.069	0.562±0.057
D	1.040±0.062	0.873±0.092	0.775±0.057	0.480±0.075
E	1.057±0.082	0.942±0.060	0.790±0.081	0.525±0.086
F	0.090±0.046	0.078±0.035	0.076±0.039	0.075±0.037

a P<0.001, compared with group B, C, D and E at the same time point.

**Table II:** Positive area ratio of vascular endothelial growth factor expression in the dura mater.

Group	1 week	2 weeks	4 weeks	12 weeks
A	0.598±0.015^a^	0.547±0.018^a^	0.400±0.014^a^	0.225±0.024
B	0.338±0.025	0.418±0.015		
C	0.350±0.029	0.425±0.016	0.338±0.027	0.223±0.024
D	0.327±0.033	0.428±0.025	0.335±0.022	0.215±0.025
E	0.342±0.029	0.422±0.012	0.327±0.019	0.218±0.023
F	0.088±0.017	0.093±0.016	0.088±0.015	0.090±0.014

a P<0.001, compared with group B, C, D and E at the same time point.
